# Evaluating the web-based ‘Partner in Balance’ program for informal caregivers of people with Huntington's disease: A pilot study

**DOI:** 10.1016/j.invent.2024.100782

**Published:** 2024-10-21

**Authors:** Maud M.J. Daemen, Lizzy M.M. Boots, Mayke Oosterloo, Marjolein E. de Vugt, Annelien A. Duits

**Affiliations:** a Department of Psychiatry and Neuropsychology / Alzheimer Center Limburg, Mental Health and Neuroscience Research Institute, Maastricht University, Maastricht, the Netherlands; b Department of Neurology, Mental Health and Neuroscience Research Institute, Maastricht University Medical Center, Maastricht, the Netherlands; c Department of Medical Psychology, Radboud University Medical Center, Nijmegen, the Netherlands; d Department of Medical Psychology, Maastricht University Medical Center, Maastricht, the Netherlands

**Keywords:** Huntington's disease, Caregiving, Support, eHealth, Self-management, Blended care

## Abstract

**Background:**

Huntington's disease (HD) poses significant challenges for both affected individuals and their informal caregivers. With the progression of HD, caregivers frequently prioritize caring for the person with HD over their own well-being. ‘Partner in Balance’ (PiB) is an 8-week online self-management program guided by a personal coach, developed to help caregivers of people with HD cope with challenging situations and develop skills to increase resilience and prevent overburdening.

**Aims:**

This pilot study evaluates the feasibility and preliminary effects of the PiB-HD program.

**Methods:**

The study employed a pre-post design. Perceived feasibility by HD caregivers was evaluated using both quantitative and qualitative measures. Preliminary effects were based on self-report measures of self-efficacy, mastery, mood, quality of life, and capability to function. Coaches' evaluations were conducted using a questionnaire.

**Results:**

In total, 18 caregivers completed the intervention. Findings demonstrate positive responses regarding the program's usability, relevance, and acceptability. Participants found the program helpful in addressing challenges, gaining insight into their actions, and feeling better equipped with skills to face future challenges. Descriptive statistics suggest that the PiB-HD program shows potential for reducing stress and anxiety. Additionally, coaches (*n* = 9) viewed the program positively for its usability, integration potential into their work, flexibility, and time efficiency.

**Conclusions:**

The PiB-HD program proved to be feasible, usable and acceptable for caregivers of people with HD. These results provide directions for further research into the effectiveness of PiB-HD, and can already be utilized to advise on the deployment of eHealth in the provision of HD care.

## Highlights

• Online self-management support feasible for informal Huntington's disease caregivers.
• Participation in Partner in Balance shows potential to reduce stress and anxiety.
• Coaches noted the program's usability, flexibility and time efficiency.
• Informal caregivers valued the coach's essential role in online support.

## 1 Introduction

Huntington's disease (HD) is a rare and hereditary neurodegenerative disorder caused by a mutation in the HTT gene, which leads to an abnormal expansion of CAG repeats (Walker, 2007). The disease follows an autosomal dominant inheritance pattern and is characterized by a triad of motor, cognitive and psychiatric symptoms (Tabrizi et al., 2019; Walker, 2007). The progression of HD is highly heterogenous, with the onset, severity, and course of symptoms differing greatly between individuals (Tabrizi et al., 2022). Beyond these symptoms, individuals with HD also face psychological challenges, such as genetic discrimination, family conflict, caregiver burden, feelings of guilt, and social isolation (Zarotti et al., 2022; Zarotti et al., 2019). Taken together, HD not only has a profound impact on the affected individual, but also on their families (Domaradzki, 2015; Parekh et al., 2018).

The progression of HD symptoms leads to an increased need for care within the home environment (Bates et al., 2015). On average, people with HD live 15 to 18 years after the first appearance of symptoms, spending about 10 years at home before transitioning to a nursing home (Ajitkumar and De Jesus, 2021; Nance and Sanders, 1996). Notably, the typical age of onset for HD falls within the range of 30 to 50 years (Roos, 2010). This early onset, together with increasing care needs of the person with HD, puts a significant pressure on family members who have to adapt to continuously changing and unpredictable circumstances.

The demanding circumstances of providing care often take their toll, leading to increased levels of emotional distress, anxiety and depression in informal caregivers of people with HD (Exuzides et al., 2022; Williams et al., 2009). Additionally, caregivers can experience role overload and feelings of isolation or loneliness (Domaradzki, 2015). Due to the hereditary nature of HD, there are added concerns that further increase the pressure on family dynamics. Parents worry about their children's risk, while children face increased anxiety over the possibility of inheriting the disease (Røthing et al., 2014; Dale et al., 2022; Vamos et al., 2007). The burden on HD caregivers has been reported to be greater in comparison to those caring for people with other neurodegenerative diseases (Mitchell et al., 2015). As a consequence, high levels of caregiver burden can compromise caregivers' ability to maintain their role as informal caregivers, which can lead to inadequate care at home, early initiation of formal home care, or premature admission to a nursing home (Roscoe et al., 2009; Aneshensel et al., 1995). Paired with the shortage of healthcare professionals and care staff, it puts an additional burden on informal caregivers at home (WHO et al., 2016).

The rising demand for home-based care highlights the necessity of supporting informal caregivers. With the progression of HD, caregivers frequently prioritize care over their own well-being (Røthing et al., 2015). This imbalance negatively affects their ability to provide good quality care – an apparent care paradox (Daemen et al., 2023). Prior research suggests helping caregivers find a better balance between caregiving and personal well-being (Domaradzki, 2015; Røthing et al., 2015; Daemen et al., 2023). Strategies that align to these needs are applied in self-management support. Such support focuses on coping with relatives' symptoms, managing their own health, and enhancing problem-solving skills (Jonkman et al., 2016; Huis in het Veld et al., 2015). Existing self-management programs have shown positive effects for caregivers of people with other neurodegenerative diseases (Huis in het Veld et al., 2015; Lyons et al., 2021). To date, no self-management program has been developed specifically for caregivers of people with HD, despite their significant caregiving burden.

An example of self-management support is the Partner in Balance (PiB) program. This blended eHealth program showed increased self-efficacy, mastery and quality of life in caregivers of people with dementia (Boots et al., 2018). PiB was also adapted and pilot tested for caregivers of people with young-onset dementia, frontotemporal dementia, and Parkinson's disease, showing positive results as well (Duits et al., 2020; Bruinsma et al., 2021a; Bruinsma et al., 2021b). Given the active phase of life of caregivers and the taboo surrounding HD, including stigma, shame, and fear of misunderstandings, these factors often discourage caregivers from seeking help. This online form of support can offer flexibility and accessibility, particularly in remote areas. Additionally, an online approach not only provides cost-effective support for HD caregivers, but also addresses disparities in post-diagnostic support access (Dorsey et al., 2018). Expanding access to support can significantly enhance the provision of HD care. Therefore, the PiB program was adapted to HD caregivers. The current study aims to evaluate the feasibility of the PiB-HD program and its preliminary effects.

## 2 Methods

The PiB program was adapted to HD caregivers (PiB-HD) following the steps of the Medical Research Council (MRC) framework (Skivington et al., 2021). This framework follows a stepwise approach: initially assessing user needs, followed by conducting a pilot evaluation to test the feasibility of the intervention and its measurement tools, and ultimately progressing to an effect evaluation. The potential user needs for PiB-HD were explored (Daemen et al., 2023) and tailored content on HD was incorporated into the existing infrastructure of PiB in close collaboration with HD caregivers, experts and healthcare professionals in HD care. HD caregivers and healthcare professionals (e.g., social workers, psychologists) identified themes that were central to the development of the program's modules (Table 1). The content, including videos, informational material, stories, and tips, was developed in collaboration with HD caregivers and healthcare professionals. Their input, including examples, personal stories, and insights from their own experiences, was used to refine and improve the content of the program. The current feasibility study employed a pre-post design to assess how caregivers of people with HD perceive the PiB-HD program.

**Table 1:** Modules in the Partner in Balance program.

Modules	Generic modules on dementia	Modules on young-onset dementia		Modules on Parkinson's disease	Modules on frontotemporal dementia	Modules on Huntington's disease
Reported in	Boots et al. (2016)	Bruinsma et al. (2021a)		Duits et al. (2020)	Bruinsma et al. (2021b)	This study
Target population	Spouses	Spouses	Other relatives	Spouses	Spouses	Spouses and other relatives
Pre-diagnostic phase						x
Future concerns						x
Nursing home admission						x
Combining work and care		x	x		x	x
Impact on family life		x	x		x	x
Sexuality and intimacy		x			x	x
Worries about heredity			x		x	x
Coping with stress⁎	x			x		
Acceptance	x	x	x	x	x	x
Balance in activities	x	x	x	x	x	x
Changes accompanying the disease	x	x	x	x	x	x
Communication	x	x	x	x	x	x
Focusing on the positive	x	x	x	x	x	x
Insecurities and rumination	x	x	x	x	x	x
Self-understanding	x	x	x	x	x	x
Social relationships and support	x	x	x	x	x	x

⁎ The module on coping with stress was merged with focusing on the positive and insecurities and rumination in the Huntington module collection.

### 2.1 The Partner in Balance program

The web-based PiB program integrates self-management principles to support informal caregivers in balancing caregiving and daily life. Guided by a healthcare professional (named ‘coach’), caregivers follow 4 online thematic modules of their own choice over 8 weeks (Table 1). Both caregivers and coaches receive a personal link to access the PiB platform. Each PiB module includes 1) a video featuring other caregivers sharing experiences, 2) psychoeducation with narrative stories, practical tips and advice, 3) a reflection assignment, and 4) a 5-step action plan. After each module, the coach provides feedback through the PiB platform to discuss and help caregivers refine their personal goals. Caregivers can also message their coach directly via the platform. The program begins with an intake to set goals and ends with an evaluation session between the caregiver and coach to discuss their experience and lessons learned for the future. This intake and evaluation could take place in person, online or over the phone, depending on personal preference.

Coaching in PiB is provided by trained healthcare professionals. Participants had the option to choose their own healthcare professional for coaching. These healthcare professionals received training, including an introductory session, e-learning, and a follow-up meeting to become familiar with PiB. The other option was to receive coaching from one of five coaches from the Maastricht University Medical Center, who have extensive experience using PiB. All coaches were contacted by the research team bi-weekly to monitor progress.

### 2.2 Participants

Participants were eligible for the study if they (1) were aged 18 years or older, (2) were an informal caregiver of a person with HD, (3) had access to the internet, and (4) were able to use a computer or tablet to access the PiB-HD program. Participants were recruited at specialized HD centers in the Netherlands, by HD healthcare professionals, and social media of the Dutch HD Association. Based on comparable feasibility studies, we aimed to include 10–15 participants (Teresi et al., 2022; Lai et al., 2013). The researchers approached all eligible caregivers, who received an information letter and were then called by the research team to address any questions. If they were willing to participate, they proceeded to complete an online informed consent. The study protocol (non-waiver) was approved by the Medical Ethics Committee of Maastricht University Medical Center (#2020–2233), the Netherlands.

### 2.3 Measurements

#### 2.3.1 Perceived feasibility by HD caregivers

Individual in-depth interviews (semi-structured) were conducted over the phone within two weeks after participants completed the program to evaluate the feasibility of PiB-HD, using the Program Participation Questionnaire (PPQ) (Boots et al., 2016). The PPQ contains 31 items on perceived usefulness, ease of use, quality of the content and overall acceptance of the program on a 7-point Likert scale ranging from 1 ‘completely disagree’ to 7 ‘completely agree’. Participants were asked to elaborate on their ratings and experiences with the program.

#### 2.3.2 Preliminary effects

A set of questionnaires was composed to examine the preliminary effects of the PiB-HD program. We also aimed to assess whether the effects of PiB-HD align with those observed in prior studies examining the feasibility and effectiveness of other versions of PiB. Therefore, participants completed a set of pre-and post-questionnaires using the same scales employed in previous studies (Boots et al., 2016; Bruinsma et al., 2021a; Duits et al., 2020; Bruinsma et al., 2021b). The questionnaire was sent online to participants within two weeks before and within two weeks after completing the program. The following scales were used: self-efficacy was measured using the Caregiver Self-Efficacy Scale (CSES), which is divided into self-efficacy care-management (5 items) and self-efficacy service use (4 items). Higher scores indicate higher levels of self-efficacy, and it has been shown to have good reliability and internal consistency (Fortinsky et al., 2002). Mastery was measured using the 7-item Pearlin Mastery Scale (PMS) (Pearlin and Schooler, 1978). The PMS has demonstrated good validity and reliability across various populations (Edwards et al., 2000; Walford-Kraemer and Light, 1984). Higher scores indicate greater mastery. Perceived stress was measured using the 10-item Perceived Stress Scale (PSS) (Cohen, 1988). Previously, the PSS demonstrated good internal consistency and validity (Andreou et al., 2011). Higher scores indicate greater perceived stress. The Hospital Anxiety and Depression Scale (HADS) (Zigmund and Snaith, 1983) was used to measure anxiety (7 items) and depression (7 items). The HADS has demonstrated good reliability and validity (Spinhoven et al., 1997), with higher scores reflecting greater levels of anxiety or depression. Quality of life was assessed using the EuroQol five dimensions questionnaire (EQ-5D) (Herdman et al., 2011). The EQ-5D has shown good validity and reliability across various conditions and populations, though some evidence of ceiling effects has been noted (Dyer et al., 2010; Pickard et al., 2008; Janssen et al., 2011). Higher scores reflect better quality of life. Lastly, capability to function was measured using the Investigating Choice Experiments for the Preferences of Older People CAPability measure for Older people scale (ICECAP-O) (Grewal et al., 2006). The ICECAP-O scale has demonstrated good construct validity and responsiveness, with higher scores indicating greater capability (Proud et al., 2019).

#### 2.3.3 Evaluation coaches

The coaches were asked to fill out a questionnaire after their participation, consisting of twelve questions. In total, 4 questions were related to the usability of the program for the coach, the possibility of integrating the program into their work, the relevance for the coach, and the relevance for the caregiver. These questions were answered on a 5-point Likert scale ranging from 1 ‘completely disagree’ to 7 ‘completely agree’. In addition, 8 open-ended questions were posed regarding program adherence, time investment, strengths and areas for improvement, and the implementation of the program within the organization.

### 2.4 Data analysis

To investigate feasibility, a total PPQ-score ranging from 31 to 217 was calculated (median = 124). Consistent with previous studies, the median score was used as a cutoff score, where a score equal to or exceeding the median was considered indicative of acceptable feasibility (Boots et al., 2016; Campbell et al., 2012). Decisions regarding positively and negatively appraised aspects of the program were based on the mean item scores, which range from 1 to 7. Mean item scores of 5 (indicating ‘slightly agree’) or higher were categorized as positive, while scores of 4 (indicating ‘slightly disagree’) or lower suggested areas needing further revision. To interpret the PPQ scores, interviews were audio-recorded and transcribed. The transcripts were analyzed using a deductive content analysis, guided by the preidentified components of the PPQ, with the analysis carried out by the first author (MD) (Elo and Kyngäs, 2008). Themes derived from the PPQ and corresponding codes were summarized into a thematic mind-map, which was subsequently discussed with the second author (LB). Following this, the findings were discussed with the entire research team for validation and finalization of the results.

For the preliminary effects, the average scores from the pre- and post-questionnaires were examined. Descriptive summary statistics, including the mean, standard deviation, and 95 % confidence intervals, were computed.

## 3 Results

### 3.1 Sample

**Fig. 1:**
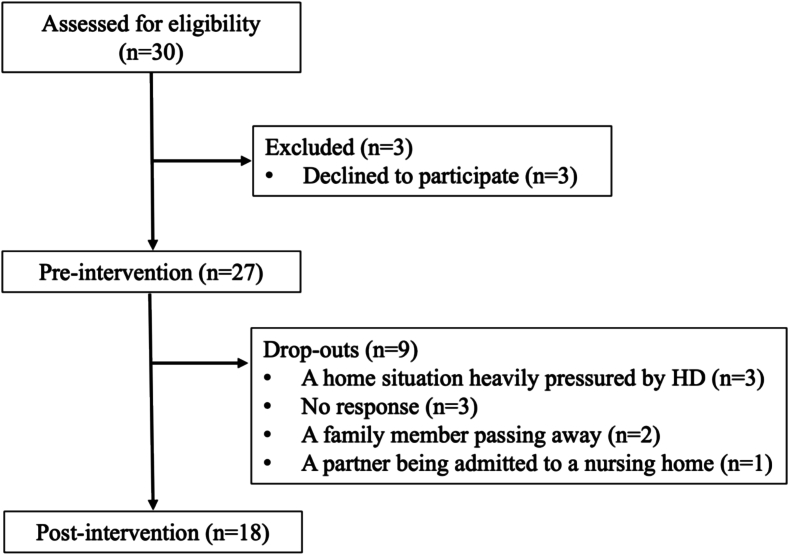
Participant flow chart.

Of those contacted by the research team, 27 out of 30 caregivers (90 %) were willing to participate (Fig. 1). The demographic characteristics of the participants are presented in Table 2. A total of 18 participants (67 %) completed the program by completing 4 selected modules and the pre- and post-evaluation questionnaires and interview.

**Table 2:** Participant characteristics.

Variable		*N* = 27
Gender	Male	7
	Female	20
Age (mean, SD)		52.4 (11.6)
Relationship to the person with HD	Spouse Brother	25 1
	Sister-in-law	1
Age of the person with HD (mean, SD)		52.8 (10.3)
Time since diagnosis	Less than 2 years	4
	Between 2 and 5 years	6
	Between 5 and 10 years	6
	More than 10 years	11
Living situation of the person with HD	Living at home, without day care Living at home, with day care	19 6
	Institutionalized	2

In total, 5 participants chose the option to be guided by a healthcare professional from the Maastricht University Medical Center because they preferred an independent perspective on their situation (*n* = 3), did not have a good relationship with their current healthcare professional (*n* = 1), or were not yet affiliated with a healthcare organization (n = 1). They were coached by a clinical neuropsychologist (n = 3), a psychologist (n = 1), or a neurologist (n = 1). The remaining 13 participants were coached by their own HD case manager or psychologist, totaling 10 different coaches, 9 of whom completed the evaluation (n = 1, no response).

### 3.2 Perceived feasibility by HD caregivers

**Fig. 2:**
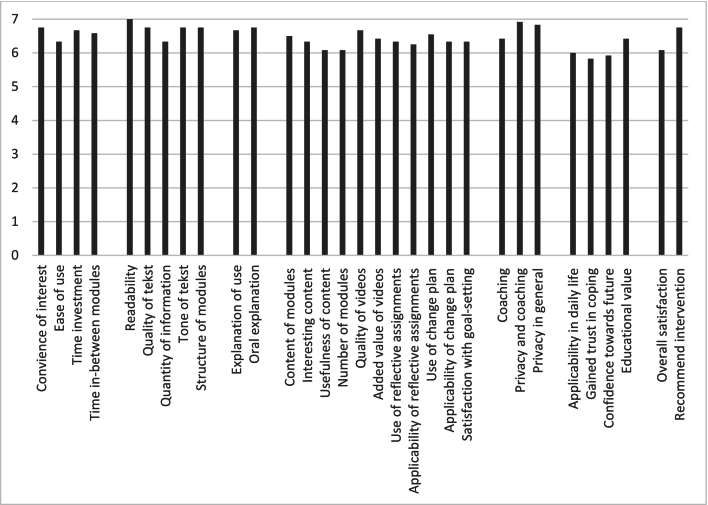
Scoring on the Program Participation Questionnaire.

After completing the program, the total sum score on the PPQ was 201.5 (SD 18.6, min. 146, max. 217), indicating good overall usability, feasibility, and acceptability since the minimum score exceeds the cut-off score of 124. All items were scored 5.8 or higher on a scale from 1 ‘completely disagree’ to 7 ‘completely agree’ (Fig. 2).

Participants indicated that there was an extensive number of modules available, ensuring that there was always something relevant to their own needs. They also valued the flexibility of the program, which allowed them to participate without specific time or location constraints, with some even citing it as a primary reason for participating as it easily fit around their working hours. The coach served as a motivating factor to engage and continue with the modules, and helped to make things specific, which participants found necessary for gaining insights or helping them to cope with challenging circumstances.

“*The coach helped me to make things specific, something I couldn't have done on my own. That specificity is necessary to act on and integrate things into your daily life. I've now made adjustments I didn't make before, things I'm truly going to keep in the future.*” – Caregiver of husband with HD (participant 3).

Participants mentioned the program has a clear and logical structure: first receiving information and then engaging in reflection and action plans. In terms of content, the videos provided recognition, which was helpful due the rare nature of HD, something participants missed in other forms of support. However, they expressed that the videos could be more extensive, with more examples so that even more people could better recognize themselves. For instance, individuals of people in later stages in nursing homes and individuals in families with young children. For individuals whose disease stage did not correspond, someone also mentioned that it helps to address things in advance for the future.

“*In terms of my situation, I think the program might have come too early for me. However, I've gone through everything with great interest, and as a result, I've thought about applying or using things later on. So, in that sense, I have a lot of tools now for the future.*” – Caregiver of wife with HD (participant 5)

The assignments and step-by-step plans proved to be useful for many participants. They noted that these assignments make things achievable, provide insight, and offer structure. As a result, they felt they could proceed working on them independently, leading to quicker action and gaining more confidence in managing the situation.

*“The past few years have been challenging and caregiving became intense. Now I feel I've been able to regain more control over the situation.”* – Caregiver of her husband with HD (participant 14)

“*The situation has not become easier, but I've gained more confidence in how to deal with the changes.*” – Caregiver of her husband with HD

(participant 9)

The assignments provided participants with tools on how to deal with certain things, but they realized that these tools set them on the right path, and it is up to them what they do with it. Participants also indicated that the program not only helps to maintain things in the short term but also focuses on improving skills for the long term, and they consciously engaged with this aspect in this program.

*“At first, I felt guilty about reducing my working hours, but not anymore. Following this program has given me the insight that it's understandable I can't keep up. Writing it down makes you aware. I've been pushing myself for so long, and now I realize I can't sustain it.*” – Caregiver of wife with HD (participant 8)

### 3.3 Preliminary effects

Pre and post assessments (mean, SD, 95 % C.I.) of all questionnaires are presented in Table 3.

**Table 3:** Scores on the pre-post questionnaires.

	Pre-intervention Mean (SD)	95 % C.I.	Post-intervention Mean (SD)	95 % C.I.
Self-efficacy care management	27.9 (6.2)	25.5–30.4	28.4 (6.3)	25.0–31.8
Self-efficacy service use	25.4 (4.3)	23.7–27.1	27.6 (6.7)	24.2–30.9
Mastery	22.5 (3.9)	21.0–24.0	23.2 (4.7)	20.9–25.5
Stress	18.1 (7.9)	15.0–21.2	13.0 (5.7)	10.2–15.8
Anxiety	8.0 (4.7)	6.2–9.9	6.4 (4.0)	4.4–8.4
Depression	6.0 (3.6)	4.5–7.4	5.3 (3.8)	3.8–7.2
Quality of Life	13.8 (0.9)	13.5–14.2	13.9 (1.1)	13.4–14.5
Capability	14.9 (2.8)	13.8–16.0	14.9 (2.1)	13.9–16.0

### 3.4 Evaluation coaches

Results showed that coaches positively evaluated the usability of the program (M = 4.0, range 1–5), the possibility of integrating the program into their work (M = 4.4, range 1–5), its relevance for informal caregivers (M = 4.3, range 1–5) and its relevance for themselves (M = 4.0, range 1–5).

Coaches were positive and satisfied with the program. They indicated PiB has a clear structure and layout. They valued the combination of receiving information through videos of peers and personal experiences, along with the stimulation provided by assignments. Aspects such as reflecting on one's own behavior and coping mechanisms, gaining self-awareness, and setting goals were seen as positive in fostering empowerment and self-reliance among informal caregivers.

*“It's remarkable how much change you can see in caregivers in a relatively short period. They manage to focus more on themselves and prioritize their own needs and interests to stay resilient.”* – Case manager

Most coaches found the program easy and flexible to use. They could access it at a time and place that was convenient for them, and they perceived the program as time-efficient. Some coaches experienced providing online feedback to be sometimes challenging because they struggled to estimate how it would be perceived by the participant. Additionally, a delayed response from the participant diminished the feeling of having a conversation and due to the online format, they felt they missed information stemming from non-verbal cues.

The majority followed the program as instructed, although some needed more than 8 weeks due to caregivers feeling overloaded or requiring extra time, such as for vacations. Coaches would highly recommend the program to other professionals, especially to support caregivers who struggle to express clear care needs or are resistant to on-site counseling. It can be less confrontational than face-to-face support while still providing valuable professional advice. Furthermore, coaches expressed interest in combining it with in-person sessions. They mentioned the desire to continue offering the program to clients in the future, highlighting the need for increased awareness within the organization to secure funding.

“*Considering the societal mission of clients living at home longer, this program adds value. Informal caregivers being more balanced means more capacity to support clients to stay at home longer.”*

– Case manager

## 4 Discussion

The blended eHealth PiB program was tailored for informal caregivers of people with HD. This pilot study aimed to evaluate the feasibility of this adapted PiB-HD program and its preliminary effects.

Overall, caregivers found the program valuable as it helped them reflect on their own needs in sustaining caregiving over time. The videos and narrative stories provided recognition and tips from others' experiences, while the assignments encouraged self-insight, helping them cope with changes in their relative with HD and their relationship. Consistent with prior research, participants specifically valued the guidance of their personal coach, including their constructive feedback, stimulation for reflection, and advice with goal-setting assignments. They also mentioned the coach as a motivating factor for engaging with and continuing the program (Boots et al., 2018; Bruinsma et al., 2021a; Bruinsma et al., 2021b). The positive PPQ responses from caregivers regarding the usability, relevance, and acceptability of the program further validate its feasibility.

Additionally, descriptive statistics suggest that the PiB-HD program shows potential for reducing stress and lowering anxiety levels. PiB aims to facilitate role adaptation, which may positively impact psychological well-being, and thus less perceived stress and anxiety (Bandura, 1997; Boots et al., 2016). Previous research on other modules of PiB for caregivers of people with dementia and Parkinson's disease did not show differences in stress levels, but did indicate a significant increase in self-efficacy (Boots et al., 2016; Bruinsma et al., 2021a; Bruinsma et al., 2021b; Duits et al., 2020). This difference can be explained by the relatively high levels of stress at baseline among HD caregivers due to their familiarity with the disease within the family and its multi-generational impact (Bayen et al., 2023; Roscoe et al., 2009). Consequently, there is relatively more room for improvement in HD caregivers, whereas stress levels for, for example, dementia caregivers were relatively low at baseline (Boots et al., 2018). Additionally, the lack of significant difference in levels of mastery in the current study might explain why there are no differences in self-efficacy outcomes, as mastery is crucial for building self-efficacy according to Bandura's social learning theory (Bandura, 1997).

From a coach perspective, the program's usability, potential for integration into their work, and relevance for informal caregivers were positively evaluated. The coaches appreciated its flexibility and time efficiency, considering it a valuable addition to supporting options for family members of people with HD. Despite some challenges with providing online feedback, coaches see potential in combining it with on-site counseling. Recognizing the crucial role of healthcare professionals in this ‘blended care’ approach (Wentzel et al., 2016; Toonders et al., 2021) underscores the need to consider their insights when integrating these support options within HD care. Furthermore, coaches highlighted the program's significance in supporting caregivers who may have limited time, encounter difficulty expressing clear caregiving needs, or are hesitant to engage in traditional face-to-face support. Improving the threshold for accessing support may effectively engage those who are typically harder to reach (Edmondson and Goodman, 2017).

Since the increase in internet access and the rise of remote services after the COVID-19 pandemic (Duits et al., 2020), online programs and telehealth interventions have become attractive options for providing support to HD caregivers (Coulson et al., 2007). This is particularly relevant because caregivers often struggle to access specific HD services due to limited availability, accessibility, and proximity, adding further strain on their time and finances (Domaradzki, 2015; Bates et al., 2015; Edmondson and Goodman, 2017). The high participation rate of 90 %, with 67 % completing the program, demonstrates a clear interest in tailored psychosocial support for HD caregivers, highlighting the unmet need for such support and the importance of further research in this area (Simpson et al., 2021). The program's flexible online use was highly valued and likely facilitated participation, with some even citing it as a primary reason for participating. Compared to other eHealth interventions, with dropout rates of up to 80 % (Geraghty et al., 2013; Donkin et al., 2011), incorporating human contact and personalization strategies in a blended format can significantly reduce dropout rates in online interventions (Atefi et al., 2024), as now exemplified by the PiB-HD program.

Altogether, the combination of quantitative and qualitative data from participants and coaches emphasized the perceived benefits of the program. However, it is important to note that these findings are based on preliminary data, so they should be interpreted with caution. Based on the MRC framework for developing and evaluating interventions (Skivington et al., 2021), these preliminary results signal the potential for further research into the effectiveness of the PiB-HD program.

### 4.1 Strengths and limitations

A strength of our study lies in its utilization of both qualitative and quantitative methods to assess caregivers' and coaches' perceptions of the PiB-HD program. Additionally, involving numerous HD healthcare organizations and professionals in the recruitment phase likely contributed to the high participation rate observed. Engaging healthcare professionals early in the process may also facilitate future implementation efforts, as these professionals gained valuable experience as coaches within the program, thereby lowering the barrier to integrating the program into daily practice (Christie et al., 2021). The quality of the coaching process and the adherence of participants and coaches are generally assessed, but both remain subjects for further research.

However, there are limitations to consider. For example, most participants are spouses of people with HD, which limits our ability to assess how adult children, siblings, or other family members who fulfill caregiving roles perceive the program. Additionally, the absence of a control condition introduces potential bias, as any observed benefits may stem from caregivers having a dedicated outlet for support and someone to talk to, rather than from the intervention itself. Furthermore, given its pilot nature, the study has limited statistical power but aims to assess the feasibility and potential effects of the PiB-HD program. Based on this, relevant outcome measures for future research on the effectiveness of the PiB-HD program can be identified. Conducting a larger-scale randomized controlled trial will be necessary to evaluate the program's effectiveness. Additionally, this study is limited to pre- and post-intervention testing, which means that the timing of completion may be influenced by daily stressors, the health status of their relative with HD, the usual care they receive, or unexpected events. Conducting multiple measurements during the intervention and including follow-up measurements in a larger controlled trial could better demonstrate the effectiveness of the PiB-HD program.

### 4.2 Clinical implications

While the effectiveness of the PiB-HD program requires validation in a larger trial, positive preliminary results from participants and coaches suggest potential for future integration into existing healthcare practices. Supported by a high participation rate and the established PiB infrastructure for informal caregivers of people with dementia and Parkinson's disease, initial preparatory steps towards implementation can be considered. Settings for implementation include, for example, nursing homes with outpatient care facilities for HD. Within these facilities, healthcare professionals play a crucial role in implementing and delivering this support program to family members. To optimize implementation, improving the self-efficacy and adherence of coaches is identified as a major facilitator (Christie et al., 2021). Therefore, healthcare professionals receive coach training consisting of online sessions and e-learning modules. Other components currently incorporated in the implementation of PiB for dementia and Parkinson's disease include inspiration and intervision sessions. These sessions, held several times a year, are designed to facilitate the exchange of insights and perspectives through peer-to-peer discussions among coaches. Both experienced and novice coaches share and reflect on their experiences with PiB, allowing lessons learned to be shared and challenges to be addressed collaboratively, such as improving the adherence of participants without applying pressure on them. Lastly, a business model has been developed to allow healthcare organizations to obtain a license to use the PiB program, to which the PiB-HD program can be added (Christie et al., 2020).

## 5 Conclusions

Our findings demonstrate the feasibility of the PiB-HD program, with positive responses regarding its usability, relevance, and acceptability. Qualitative results show that participants indicated the program to be helpful in addressing challenges, gaining insight into their actions, building confidence in managing their situations, and feeling better equipped with skills to face future challenges. Descriptive statistics suggest that the PiB-HD program shows potential for reducing stress and lowering anxiety levels. Furthermore, coaches who guided the informal caregivers within the program had a positive view of the program's usability, potential for integration into their work, relevance for informal caregivers, flexibility, and time efficiency.

Notably, the current study is a pilot study which does not allow for conclusions regarding the effectiveness of the PiB-HD program. However, the positive feasibility results do provide direction for further research into its effects. The findings of this current study can already be utilized to advise on the deployment of eHealth in the provision of HD care.

## Funding

The project was funded by the European Huntington's Disease Network [EHDN]. Grant number 1137.

## Declaration of competing interest

The authors declare the following financial interests/personal relationships which may be considered as potential competing interests: Maastricht University and the Academic Hospital Maastricht, with Mayke Oosterloo as inventor, have submitted a patent application #EP23197746. The other authors have no conflict of interest to report.
